# Consensus on β-Lactamase Nomenclature

**DOI:** 10.1128/aac.00333-22

**Published:** 2022-04-05

**Authors:** Patricia A. Bradford, Robert A. Bonomo, Karen Bush, Alessandra Carattoli, Michael Feldgarden, Daniel H. Haft, Yoshikazu Ishii, George A. Jacoby, William Klimke, Timothy Palzkill, Laurent Poirel, Gian Maria Rossolini, Pranita D. Tamma, Cesar A. Arias

**Affiliations:** a Antimicrobial Development Specialists LLC, Nyack, New York, USA; b Cleveland VA, Cleveland, Ohio, USA; c Indiana University, Bloomington, Indiana, USA; d Sapienza University of Rome, Rome, Italy; e The National Center for Biotechnology Information, National Library of Medicine, National Institutes of Health, Bethesda, Maryland, USA; f Toho University School of Medicine, Tokyo, Japan; g Lahey Hospital and Medical Center, Burlington, Massachusetts, USA; h Baylor College of Medicine, Houston, Texas, USA; i Emerging Antibiotic Resistance, Medical and Molecular Microbiology, Department of Medicine, University of Fribourg, Fribourg, Switzerland; j Swiss National Reference Center for Emerging Antibiotic Resistance, Fribourg, Switzerland; k INSERM European Unit (LEA), (IAME, Paris) University of Fribourg, Fribourg, Switzerland; l University of Firenze, Florence, Italy; m Microbiology and Virology Unit Careggi University Hospital, Florence, Italy; n Johns Hopkins University School of Medicine, Baltimore, Maryland, USA; o Division of Infectious Diseases, Houston Methodist Hospital, Houston, Texas, USA; p Center for Infectious Diseases Research, Houston Methodist Research Institute, Houston, Texas, USA

**Keywords:** beta-lactamases

## Abstract

Assigning names to β-lactamase variants has been inconsistent and has led to confusion in the published literature. The common availability of whole genome sequencing has resulted in an exponential growth in the number of new β-lactamase genes. In November 2021 an international group of β-lactamase experts met virtually to develop a consensus for the way naturally-occurring β-lactamase genes should be named. This document formalizes the process for naming novel β-lactamases, followed by their subsequent publication.

## TEXT

Naming of β-lactamases has been approached in a variety of ways over the past 4 decades. Prior to 1997, all β-lactamases including laboratory constructs, were named by the individuals who first described these unique β-lactam-hydrolyzing enzymes, sometimes ignoring the exact nature of the protein or its corresponding gene at the molecular level. The first β-lactamase names often reflected the bacterial species, strain, plasmid, or patient from which the enzymes were isolated (1). Later, enzyme names were related to substrates hydrolyzed, inhibitors affected, biochemical properties, sequence anomalies, geographical location of their discovery, and, occasionally, on the investigators who described them (1). In addition, the characterization of new β-lactamase enzymes was based upon their spectrum of activity and iso-electric point. This random, and sometimes whimsical approach, was adequate for the few dozen β-lactamases recognized by the mid-1980s. However, the use of expanded-spectrum cephalosporins began to select for the now common extended-spectrum β-lactamases (ESBLs), and new TEM and SHV variants emerged rapidly and new enzymes with ESBL activity were became more common in isolates circulating in the clinical setting (2, 3). During this time, nucleotide sequencing became more accessible and inexpensive, and molecular descriptions of novel *bla* alleles were included in many publications. The result was an explosion of new β-lactamases with sometimes overlapping and poorly defined names (4). Multiple names published either for the same enzyme, e.g., TEM-3/TEM-14 or TEM-12/YOU-2/CAZ-3/TEM-E2, or group of enzymes (e.g., GES/IBC or CTX-M/FEC/MEN/TOHO) or the same name was assigned to different enzymes in different journals (1). Perhaps most distressingly, little attention was paid to amino acid sequence identities within families.

In an attempt to bring consistency to the confusion, a group of β-lactamase investigators met following a β-lactamase symposium at the 1996 ICAAC meeting. At that time, a consensus was reached on the following points:

- George Jacoby, the Editor-in-Chief of *Antimicrobial Agents & Chemotherapy* (AAC) at the time, would establish a nomenclature reference site at a Lahey Clinic studies site (lahey.org/studies/), initially serving to assign names to TEM, SHV and OXA extended-spectrum and inhibitor resistant enzymes.
- Any naturally occurring β-lactamase with a novel amino acid sequence would be assigned a new number, with an effort to lump members of closely related molecular structures into the same family.
- Instructions to Authors for ASM journals would require new β-lactamase numbers to be vetted by the Lahey curators before publication in the journal beginning in 1997.

The Lahey Clinic studies site eventually included compilations of all the amino acid sequences for TEM and SHV enzymes, with documentation as to GenBank accession numbers, alternate names, isoelectric points and publication information. Names were later assigned to other β-lactamases in all the major families, including those in classes B, C and D. Karen Bush and Timothy Palzkill were later added as co-curators. Other websites cited on the Lahey site have compiled data for TEM, SHV, and class B enzymes at http://www.laced.uni-stuttgart.de, and https://bigsdb.pasteur.fr/klebsiella/ for LEN, OXY, and OKP β-lactamases. The Lahey site was maintained through June 2015, and remains archived online with historical data still available.

Until the early 2000s, almost all publications characterizing novel β-lactamases included functional information, with full nucleotide and amino acid sequences becoming a standard descriptor. However, as whole genome sequencing (WGS) became more accessible, historical collections of dozens of clinical isolates were examined for any nucleotide sequence that resembled a β-lactamase, regardless of proof of functionality. The curators realized that many thousands of new β-lactamase sequences potentially could be identified from natural isolates, as predicted by the Palzkill laboratory in the 1990s, when it was observed that mutations at all but 43 of the 263 amino acids in the TEM-1 sequence could result in a functional β-lactamase (5). Thus, it was feared that future WGS campaigns might easily identify an unworkable number of new β-lactamase-like sequences. Because the workload was already becoming challenging for the curators to provide new numbers in a timely manner, a larger scale operation was necessary. Discussions were initiated with Michael Feldgarden and William Klimke of The National Center for Biotechnology Information (NCBI) prior to ICAAC in 2014, resulting in three-way conversations among NCBI personnel, Lahey site curators, and AAC journal staff. The groups mutually decided that assignment of new β-lactamase allele numbers would be transferred to NCBI beginning in July 2015. All data from the Lahey site were then transferred to NCBI where the same considerations were retained as for the Lahey site, namely, that β-lactamase numbers would be assigned based on amino acid sequences, and that only naturally-occurring β-lactamases (as opposed to laboratory-generated mutants) could receive a new number.

NCBI assignment of β-lactamase names has continued to proceed successfully, with this convention generally followed by AAC. However, several new β-lactamase allele numbers were recently appropriated by non-AAC authors without NCBI consultation that conflicted with existing allele designations. For example, we noticed recently two different usages of the allele assignment *bla*_NDM-16_. The more common allele was discussed critically in two publications, Li et al. (6) and Cheng et al. (7), one of which had detailed functional characterization. The other assignment had granted previously by the Lahey Clinic site (8). After review of the literature and consultation with recent submitters, we updated the allele assignments for these two proteins in NCBI’s Reference Gene Catalog and, with the submitters’ permission, the associated GenBank records. The original Lahey-assigned NDM-16 (AKZ20823.1) is now NDM-16a, whereas BCO01847.1 is now NDM-16b, with NDM-32 as a synonym. Despite this work, the published literature still contains incorrect assignments, potentially leading to confusion. We would note that these conflicts unfortunately are not unique to β-lactamases, as they have occurred with other AMR genes, such as MCR (mobile colistin resistance) which led to the creation of an MCR nomenclature scheme (9).

Because these problems are not uncommon, we propose that all ASM journals, and other journals that publish β-lactamase articles, adopt the following guidelines as recently discussed by a community of β-lactamase investigators. Experts representing 19 countries were invited to participate in a virtual workshop hosted by ASM (supplemental table). During the meeting, participants were able to review the proposed rules and weigh in on various aspects during breakout sessions. The rules and proposed update to the Instructions to Authors is a consensus of the views expressed during the meeting. We recommend that editors and reviewers to be diligent in maintaining consistency in the naming of these important enzymes.

## IMPORTANT RULES GOING FORWARD

- New β-lactamase numbers will continue to be assigned based on predicted amino acid sequences that differ by at least one amino acid substitution from previously designated sequences.
  - New allele requests can be based on predicted amino acid changes in the leader sequence.
  - DNA sequences that differ by a single nucleotide that does not encode a different amino acid (synonymous change) will not be given a new allele number.
  - Only β-lactamases from natural sources will be numbered, not laboratory constructs.
- Allele numbering for most β-lactamase families is designated and tracked by NCBI and new allele numbers of these gene families will be assigned by NCBI if necessary (if a submitted but not yet published allele number exists, and it matches the new submitted sequence, that allele number will be assigned). The list of families for which NCBI assigns alleles can be found on this page: https://www.ncbi.nlm.nih.gov/pathogens/submit-beta-lactamase/.
- Allele requests should be made only when constructive, that is, where single amino acid changes likely will have functional or epidemiological significance, and where citing allele numbers provides greater clarity than simply citing protein accession numbers.
- For large families of chromosomal class C β-lactamases (*ampC*) that so far have not received extensive allele assignments, allele numbers may not be assigned. The *bla*_ADC_, *bla*_ACT_, and *bla*_PDC_ families are exceptions for which allele assignments are well-established and new requests are welcome.
- The nomenclature for OXA-type β-lactamases is in evolution and efforts are underway to meet this challenge in a subsequent publication.
- New β-lactamases should not be named based on geographical location.
- The proper format for citations of β-lactamase genes should be written following the convention for *bla*_TEM-1_ (italicized *bla*, followed by subscript allele designation).
- Functional categorization of β-lactamases will not be assigned based upon sequence similarity (i.e. ESBL phenotype cannot be based on sequence alone).

## UPDATED INSTRUCTIONS TO AUTHORS FOR ASM JOURNALS

To determine if an allele is novel, submitters should review the list of existing alleles in the Reference Gene Catalog (https://www.ncbi.nlm.nih.gov/pathogens/refgene/). If the predicted protein sequence is novel, they should proceed with the following submission procedure:

The complete nucleotide sequence and the complete protein translation from the nucleotide sequence, including the signal peptide for a novel allele must be submitted to the International Nucleotide Sequence Database Collaboration (INSDC) which includes GenBank, DNA Databank of Japan (DDBJ) and the European Nucleotide Archive (ENA) and the Accession Number referred to in the manuscript. Novel β-lactamase gene names and new allele numbers of existing β-lactamase families are assigned by NCBI. Information on submitting a request for a new β-lactamase can be found at https://www.ncbi.nlm.nih.gov/pathogens/submit-beta-lactamase/. The curators of that site must be consulted regarding the name of a potentially novel β-lactamase sequence before a new number designation is proposed for publication. Curators also will answer questions about what may be submitted or the submission process when contacted at pd-help@ncbi.nlm.nih.gov. Written confirmation of the approved name designated by NCBI should be provided with manuscript submission.

*If you have previously submitted a sequence to GenBank (*e.g., *a draft genome*): please contact the curators at pd-help@ncbi.nlm.nih.gov with the protein accession of the sequence, and curators will assign a novel allele designation, if needed.

*If you have an unsubmitted sequence:* For novel β-lactamase protein sequences (i.e., those not publicly available in GenBank), users must submit the sequence to GenBank following the instructions on the Pathogen Detection website (https://www.ncbi.nlm.nih.gov/pathogens/submit-beta-lactamase/).

The style used to refer to β-lactamase genes will follow the format of italicized *bla*, followed by subscript allele designation (e.g., *bla*_TEM-1_). When referring to β-lactamases, use the functional designations defined by Bush and Jacoby (10), in addition to the molecular classification as initiated by Ambler (11). Biochemical studies performed to characterize the function of a β-lactamase or the interaction of a compound with a β-lactamase (i.e., as a substrate, or inhibitor) should follow the guidelines set forth by Bush and Sykes (12). Biochemical characterizations should preferably be performed on a purified enzyme particularly with regard to defining the substrate profile. Alternatively, a cellular extract that contains a single β-lactamase activity can be used for certain minimal parameters. *K_m_* and *V_max_* can be reported for periplasmic extracts but *k_cat_/K_m_* can only be reported for purified enzymes (>95% purity as determined by SDS-PAGE using 20 μg of protein or by mass spectrometry). Hydrolysis studies should include at least two substrates appropriate for defining the functional activity of the enzyme (e.g., cefotaxime and ceftazidime hydrolysis for extended-spectrum β-lactamases/ESBLs and expanded spectrum AmpCs).

Inhibition data should be reported as *K_i_* values for reversible inhibitors in the presence of a stated concentration of substrate whose kinetic parameters have been defined. An efficiently hydrolyzed substate such as nitrocefin should be used. For irreversible inactivators, IC_50_ values may be reported, accompanied by sufficient experimental details regarding incubation times and substrate concentration. Reproducibility of results from at least three different determinations must be shown. If substrate profiles of two enzymes are compared, both enzymes must be studied under identical conditions. When testing metallo-β-lactamases, the amount of zinc supplemented to the assay mixture must be specified. For the testing of OXA β-lactamases, the bicarbonate concentration in the assays must be provided.

Minimal functionality assessments may be based on MIC determinations that are performed by reference broth or agar dilution tests. Strains used for MICs must include a cloned enzyme that contains the original leader sequence expressed from a β-lactamase-free vector in hosts whose genetic background lack any detectable β-lactamase gene expression. For comparison, MIC determinations must also include the host strain without any β-lactamase, a plasmid vector control and the original clinical isolate.
